# *Salmonella* Biofilm Formation, Chronic Infection, and Immunity Within the Intestine and Hepatobiliary Tract

**DOI:** 10.3389/fcimb.2020.624622

**Published:** 2021-02-02

**Authors:** Jaikin E. Harrell, Mark M. Hahn, Shaina J. D’Souza, Erin M. Vasicek, Jenna L. Sandala, John S. Gunn, James B. McLachlan

**Affiliations:** ^1^Department of Microbiology and Immunology, Tulane University School of Medicine, New Orleans, LA, United States; ^2^Center for Microbial Pathogenesis, Abigail Wexner Research Institute at Nationwide Children’s Hospital, Columbus, OH, United States; ^3^Infectious Diseases Institute, The Ohio State University, Columbus, OH, United States; ^4^Department of Pediatrics, College of Medicine, The Ohio State University, Columbus, OH, United States

**Keywords:** biofilm, chronic infection, immunity, hepatobiliary, *Salmonella*

## Abstract

Within the species of *Salmonella enterica*, there is significant diversity represented among the numerous subspecies and serovars. Collectively, these account for microbes with variable host ranges, from common plant and animal colonizers to extremely pathogenic and human-specific serovars. Despite these differences, many *Salmonella* species find commonality in the ability to form biofilms and the ability to cause acute, latent, or chronic disease. The exact outcome of infection depends on many factors such as the growth state of *Salmonella*, the environmental conditions encountered at the time of infection, as well as the infected host and immune response elicited. Here, we review the numerous biofilm lifestyles of *Salmonella* (on biotic and abiotic surfaces) and how the production of extracellular polymeric substances not only enhances long-term persistence outside the host but also is an essential function in chronic human infections. Furthermore, careful consideration is made for the events during initial infection that allow for gut transcytosis which, in conjunction with host immune functions, often determine the progression of disease. Both typhoidal and non-typhoidal salmonellae can cause chronic and/or secondary infections, thus the adaptive immune responses to both types of bacteria are discussed with particular attention to the differences between *Salmonella* Typhi, *Salmonella* Typhimurium, and invasive non-typhoidal *Salmonella* that can result in differential immune responses. Finally, while strides have been made in our understanding of immunity to *Salmonella* in the lymphoid organs, fewer definitive studies exist for intestinal and hepatobiliary immunity. By examining our current knowledge and what remains to be determined, we provide insight into new directions in the field of *Salmonella* immunity, particularly as it relates to chronic infection.

## Introduction

Most bacteria can grow as either individual planktonic cells or as communities within a biofilm (Flemming and Wuertz, 2019). Biofilms are surface-attached aggregates of cells surrounded by a self-produced extracellular polymeric substances (EPSs) (Costerton et al., 1999; Branda et al., 2005; Hall-Stoodley and Stoodley, 2009; Flemming, 2016). The development of these structures often occurs in response to perceived negative environmental stimuli such as shifts in pH, temperature, oxygen, and nutrient availability (O’Toole and Kolter, 1998; O’Toole et al., 2000a; O’Toole et al., 2000b). Individual cells adhere to a surface, biotic or abiotic, producing EPSs such as proteins, exopolysaccharides, and nucleic acids. In this niche, they grow and mature, at which point some cells detach and return to planktonic growth to repeat the process (Khatoon et al., 2018). While the bacteria reside within a biofilm they are protected from a variety of challenges such as UV exposure (Espeland and Wetzel, 2001), host defense mechanisms, and antibiotics (Mah and O’Toole, 2001; Stewart and Costerton, 2001). As a result, biofilms play an essential role in the environment, industry, and medicine.

In the natural environment, biofilms are important for microbial survival. Biofilms provide a stable environment for the bacteria by fostering symbiotic relationships with other organisms, such as settlement of marine invertebrate larvae (Nelson et al., 2020), degrading harmful chemicals in the soil (Ahmad et al., 2017), and promoting plant growth (Liu et al., 2013). However, biofilms are less desirable in industries such as food, water, energy, and medicine, where they can cause harm by developing on food and food processing equipment (Flemming, 2016; Galie et al., 2018), contaminating water pipelines (Wingender and Flemming, 2011), corroding underwater metal surfaces (de Carvalho, 2018), and persisting on medical devices (Percival et al., 2015).

Here we focus on biofilms in the field of medicine. Biofilm communities can enhance recalcitrance to host defense mechanisms. As a result, biofilms are estimated to be involved in approximately 80% of chronic infections, which increase hospitalization rates, healthcare costs, and morbidity and mortality (Davies, 2003). Examples of biofilm-associated diseases include several respiratory diseases, chronic otitis media, periodontitis, and chronic wound infections (Høiby et al., 2010; Wessman et al., 2014; Bao et al., 2015; Wu et al., 2015). In cystic fibrosis (CF), persistent lung infections are often caused by *Pseudomonas aeruginosa* biofilms or aggregates. Due to the ability of the biofilm to resist phagocytosis *via* polysaccharide synthesis locus (Psl) (Mishra et al., 2012) and other EPS components, chronic inflammation causes lung tissue damage (Høiby et al., 2010). Additionally, CF patients co-infected with *Staphylococcus aureus* have been shown to have a greater decline in lung function than when infected with *P. aeruginosa* alone (Limoli et al., 2016; Maliniak et al., 2016; Limoli and Hoffman, 2019). Such interspecies interactions alter the efficacy of antibiotics and lead to worse patient outcomes.

Biofilms also develop on abiotic medical devices such as prosthetics, stents, catheters, implants, and dentures (Percival et al., 2015). Biofilms on indwelling medical devices are estimated to cause 50% of persistent nosocomial infections (Paredes et al., 2014). The most commonly identified biofilms are formed by *Staphylococcus epidermidis*, *S. aureus*, and *P. aeruginosa* (Hall-Stoodley et al., 2004; Khatoon et al., 2018). *Staphylococcus epidermidis* is the model bacterial species for dental implant-related biofilms, which are different than the biofilms that form on teeth (Daubert and BF, 2019). The shape of the dental implant makes complete elimination almost impossible (Müsken et al., 2018). The bacteria that survive treatment result in persistent biofilm infections, implant failure, and promote the development of antibiotic resistance.

Multidrug resistant bacteria have become a serious medical concern, accounting for over 2.8 million infections and 35,000 deaths annually in the United States (CDC, 2019). Bacteria residing within a biofilm are also recalcitrant to antibiotics, surviving levels 10–1,000 times greater than planktonic cells (Høiiby et al., 2010; Mah, 2012). The close association of bacteria in a biofilm can also promote antibiotic gene transfer (Christensen et al., 1998; Hausner and Wuertz, 1999; Li et al., 2018). Biofilm recalcitrance to antibiotics is due to complex factors that are incompletely understood and often involve multiple species, which limits the efficacy of antibiotic clearance (Jiang et al., 2020). While improvements have been made in preventing biofilm formation, such as the development of medical devices made or coated with antibacterial materials (Vasilev et al., 2018), surgical removal of the infected tissue or medical device is often required for complete resolution (Høiiby et al., 2010).

Infections due to biofilm-associated bacteria are a significant clinical problem. Therefore, further studies are needed to identify the key aspects of biofilm formation and persistence in order to develop novel preventative and therapeutic strategies to enhance the efficacy of treatment.

## Biofilms and Chronic Infections

Whether comprised of a single species or polymicrobial, biofilms demonstrate the remarkable ability of bacteria to coordinate functions and develop complex behaviors. While the discovery of biofilms coincides with the discovery of microorganisms by Antoni van Leeuwenhoek in 1683, this unique modality of growth was originally overlooked in a medical context, gaining prominence only in the past 50 years. However, the study of infectious biofilms has overwhelmingly demonstrated their important role in chronic infections, with, as mentioned previously, estimates by the National Institutes of Health that 80% of all chronic infections are related to biofilms (Davies, 2003).

Whether opportunistic or primary pathogens, many bacteria are capable of causing acute or chronic infections, depending on circumstance. While we do not wish to downplay the medical importance of acute infections, we will mention them only briefly in this review as our focus is chronic infections. Acute infections coincide with rapid growth and dissemination in the host leading to overt clinical signs days after onset of disease (Furukawa et al., 2006; Thakur et al., 2019). Acute infections generally lead to one of three consequences: successful clearance (either naturally by the immune system or with medical intervention), death of the host, or development into a latent infection. In this third outcome, the acute phase of infection is followed by a dormant phase with long-term infection that can span the life of the host and is characterized by repeated spells of reactivation (Thakur et al., 2019). This is a type of chronic infection. Reactivation is associated with production of infectious agents that can be transmitted to others although this activity may or may not renew symptoms in the host (Thakur et al., 2019).

While latent infections only develop after an initial acute phase, many pathogens do not cause acute disease and in these cases may directly result in a chronic infection. These chronic infections can arise opportunistically from a compromised immune system, an altered microbiota, a breach in skin or mucosal immune barriers, or from contamination at surgical sites and on implanted medical devices (Furukawa et al., 2006; Thakur et al., 2019). Both bacteria and host responses to their environment can result in genetic or phenotypic alterations that foster chronic infection. Several recent reviews have catalogued various chronic infections by body site (Vestby et al., 2020), organism and immune response (Thakur et al., 2019), or survival strategy (facultative intracellular, small colony variants, persister populations, environmentally induced antibiotic indifference, and biofilm formation) (Grant and Hung, 2013).

A common theme of chronic infections is their ability to last at least a few months, often years, and even up to the entire lifetime of the host. While a long-term infection in the absence of acute disease is the most rudimentary definition, other characteristics are fundamental to chronic infections. Similar to latent infections, chronic infections are generally asymptomatic and usually do not pose immediate risk to host health (Grant and Hung, 2013; Thakur et al., 2019). However, the possibility of future reactivation of latent/chronic infections into clinically significant disease, disease dissemination, or the onset of malignancy all pose long-term risks (Grant and Hung, 2013). There is often a direct link between chronic infections and biofilm formation (Parsek and Singh, 2003; Hall-Stoodley et al., 2004; Furukawa et al., 2006) as long-term survival requires pathogen stealth and/or a protected niche for the pathogen. Both properties are enhanced by biofilms and while the niche could be provided by granulomas or intracellular compartments, survival in these locations is associated with slowed growth and reduced replicative capacity. However, unlike latent infections, chronic infections often have a high level of bacterial replication and concomitant burden of the pathogen (albeit less than acute infection) (Furukawa et al., 2006; Thakur et al., 2019). This activity is possible by forming a biofilm, which alters the bacterial growth physiology to allow for significant tolerance to elevated levels and prolonged administration of antibiotics as well as tolerance to other host responses such as the complement system, antimicrobial peptides, antibodies, and phagocytic activity by neutrophils and macrophages as further discussed below (Furukawa et al., 2006; Grant and Hung, 2013; Pletzer et al., 2017; Thakur et al., 2019). Furthermore, biofilm formation permits shedding of planktonic bacteria and/or biologically active molecules into the host (Zegans et al., 2002; Furukawa et al., 2006) which has pleiotropic effects on the host immune state and is important for maintaining a favorable niche by altering the activation of innate immune receptors, inhibiting apoptosis, inducing an inappropriate immune response, or causing immunosuppression (Thakur et al., 2019). Chronic infections often maintain their niche by simultaneous activation of both the innate and adaptive immune responses (Vestby et al., 2020), but interestingly, neither of these responses eliminate the pathogen but instead result in collateral damage to host cells and tissue (Moser et al., 2017; Vestby et al., 2020). Accumulation of macrophages and neutrophils at the site of infection is characteristic of chronic infection (Laskin et al., 2011; Pletzer et al., 2017). Prolonged reactive oxygen species and reactive nitrogen species production by these cells represent a significant source of inflammation (Grant and Hung, 2013; Mittal et al., 2014; Pletzer et al., 2017). This activity ultimately benefits the pathogen as chronic inflammation from an aberrant immune response makes host nutrients available to the biofilm (Pletzer et al., 2017; Thakur et al., 2019), obstructs would healing, and facilitates cellular invasion for dissemination, access to other protected sites, or acute infection (Vestby et al., 2020). Chronic inflammation is also linked to many malignancies associated with chronic infection, including gastric adenocarcinoma and gastric lymphoma from *Helicobacter pylori* infections (Wotherspoon et al., 1991; Nomura et al., 1994) or gallbladder carcinoma from *Salmonella* Typhi infections (Caygill et al., 1994; Grant and Hung, 2013; Vestby et al., 2020).

To highlight an example, *P. aeruginosa*, mentioned earlier, is well-suited for opportunistic chronic infections. While ubiquitous in soil and water and a common colonizer of animals and humans, *P. aeruginosa* rarely infects healthy individuals but can infect multiple body sites in the immunocompromised (Wu and Li, 2015). While *P. aeruginosa* may have the most notoriety for infecting CF patients, it is also consequential in ventilator-associate pneumonia (Bergmans and Bonten, 1999; Mulcahy et al., 2014), on orthopedic implants and joint replacement surgical sties (Mulcahy et al., 2014), catheters (Cole et al., 2014), and in severe soft tissue wounds (Lochab et al., 2020). Characteristic of chronic biofilm infections, *P. aeruginosa* infections are difficult to detect with clinical microbiology techniques (Costerton et al., 2003; Mulcahy et al., 2014) and are extremely recalcitrant to antibiotics and the host environment (Mulcahy et al., 2014; Lochab et al., 2020). In 2015, hospitalized cancer, CF, or burn wound patients with *P. aeruginosa* infection experienced a 50% mortality rate (Gómez and Prince, 2007; Wu and Li, 2015) and the CDC reports that *P. aeruginosa* is the fourth-most isolated pathogen in hospitals, accounting for 10% of all nosocomial infections (Wu and Li, 2015). There is ample evidence to conclude *P. aeruginosa* relies on a biofilm forming strategy for survival in the host and that *P. aeruginosa* biofilms control the host response to establish a favorable environment. In most instances of *P. aeruginosa* disease, the sustained collateral damage from frustrated neutrophil phagocytosis at the site of biofilm infection is the primary cause of major disease sequellae (Jensen et al., 2010; Mulcahy et al., 2014). Remarkably, studies of CF infants have demonstrated *P. aeruginosa* biofilms remodel host immunity from a balanced Th1/Th2 response to a Th2 response with reduced levels of IFN-γ (Moser et al., 2000; Mulcahy et al., 2014).

*Salmonella* Typhi is an especially interesting pathogen because of its ability to cause disease in all three mechanisms discussed. As the primary etiologic agent of typhoid fever (Stanaway et al., 2019) (a systemic disease that can be deadly) the organism must be considered an acute pathogen. However, the only known reservoir of *S*. Typhi is human carriers who have long-term infections primarily by biofilms on cholesterol gallstones in the gallbladder. Interestingly, about 5% of acute infection survivors will develop latent infections but as many as 25% of chronic carriers have gallstone biofilms without ever expressing symptoms of acute disease (Parry et al., 2002; Gonzalez-Escobedo et al., 2010; Gunn et al., 2014). These different disease strategies (acute, latent, and chronic) highlight the complex nature of host-pathogen interactions during chronic infection.

## *Salmonella* Chronic Infections

In addition to *S*. Typhi, many other serovars of *Salmonella enterica* possess the ability to colonize and potentially cause disease in humans and/or other animal hosts. Biofilm formation in these serovars is highly conserved—especially in the serovars that are able to colonize multiple hosts—suggesting that the ability to form a biofilm serves as an evolutionarily advantage during the cycle of infection and transmission (Römling et al., 2003; MacKenzie et al., 2017; MacKenzie et al., 2019). As with other biofilm-forming bacteria, biofilm-associated *Salmonella* are encased within a matrix of EPSs consisting of proteins, carbohydrates, and extracellular DNA (eDNA) (Maruzani et al., 2019). The exact composition of the *Salmonella* biofilm EPSs may vary depending on both serovar and environmental conditions, but primarily consists of a network of proteinaceous curli fimbriae fibers, cellulose, and eDNA (Zogaj et al., 2001; Römling, 2005; Wang et al., 2014). Additional EPS components may include cellular appendages such as flagella or various adhesive fimbriae (Römling and Rohde, 1999; Boddicker et al., 2002; Prouty et al., 2002; Prouty and Gunn, 2003; Ledeboer et al., 2006; Dwyer et al., 2011); cell surface proteins such as the large biofilm-associated surface protein BapA (Latasa et al., 2005); and other exopolysaccharides such as colanic acid or the O-antigen capsule (Ledeboer and Jones, 2005; Crawford et al., 2008). A self-secreted “common good,” these EPSs collectively allow cells to securely adhere to both surfaces and other cells within the biofilm, help retain moisture in dry environments, and possibly slow the diffusion of harmful molecules such as antimicrobial peptides and antibiotics (Dieltjens et al., 2020).

The transition of planktonic *Salmonella* into a biofilm state may be influenced by a variety of environmental triggers, including temperature fluctuations, changes in nutrient availability, and exposure to harsh or harmful substances (De Oliveira et al., 2014; Paytubi et al., 2017; González et al., 2019b). Accordingly, the regulatory network controlling the expression of biofilm genes is highly complex. Numerous global regulators, two-component response systems, and other regulatory proteins and small RNAs contribute to regulate expression of key biofilm genes, primarily through expression of *csgD* (Simm et al., 2014). An orphan response regulator encoded within the curli biosynthesis operon, CsgD is often regarded as the master biofilm regulator in *Salmonella* (Gerstel and Römling, 2003). CsgD induces the production of the two major EPS components, curli fimbriae and cellulose, by directly binding the *csgB* promoter within the *csgBAC* curli biosynthesis operon and by inducing expression of the diguanylate cyclase AdrA, which subsequently regulates cellulose biosynthesis through the modulation of the secondary messenger (3’-5’)-cyclic-diguanosine monophosphate (c-di-GMP) (Römling et al., 2000; Zakikhany et al., 2010). Evidence suggests CsgD may also regulate the expression of other EPS genes, including those responsible for the production of BapA and the O-antigen capsule (Latasa et al., 2005; Gibson et al., 2006).

Outside of the host, biofilm formation allows for *Salmonella* to attach to a variety of biotic and abiotic surfaces, thereby enabling the bacteria to persist in a viable but relatively dormant state until ultimately ingested by a host. Because *Salmonella* are enteric pathogens, non-typhoidal *Salmonella* biofilms are of particular concern in the agricultural and food processing and packaging industries where they contaminate both fresh and processed food products (Yaron and Römling, 2014; Pande et al., 2016; Galie et al., 2018; Lamas et al., 2018; Merino et al., 2019). Contamination of food products with *Salmonella* typically originates from colonized or infected livestock including chickens, pigs, and cattle (Lamas et al., 2018). This contamination may be direct, e.g., if the intestinal contents of infected animal carcasses are released during processing, but is more often indirect, occurring when uncompromised products come into contact with previously contaminated processing surfaces or machinery (Reij and Den Aantrekker, 2004; Giaouris et al., 2012). *Salmonella* has been shown to adhere as a biofilm to multiple abiotic materials used in industrial settings, including stainless steel, polystyrene, and glass (Joseph et al., 2000; Giaouris and Nychas, 2006; Chia et al., 2009; Kim and Wei, 2009). While sanitation practices are implemented to disinfect potential sources of cross-contamination, *Salmonella* within a biofilm are much more recalcitrant to disinfectants and other antimicrobials compared to their planktonic counterparts, rendering such practices ineffective (Joseph et al., 2000; Sheffield et al., 2009; Corcoran et al., 2014; Galie et al., 2018). Additionally, after a product has been contaminated, *Salmonella* can also form a biofilm directly on the surface of the food itself. Raw meat and poultry, fresh fruits and vegetables, and even low-moisture processed foods such as cereal products have been shown to support *Salmonella* biofilm growth, supporting the theory that biofilm formation is a critical method of persistence outside of the host (Tamblyn et al., 1997; Podolak et al., 2010; Yaron and Römling, 2014; Lamas et al., 2018).

## *Salmonella* Biofilms Within the Infected Host

While the association of the *Salmonella* biofilm phenotype with persistence outside of the host has long been established, additional research was required to fully interrogate the role of *Salmonella* biofilms in chronic infection within the host. While many researchers originally doubted biofilm formation was possible *in vivo* due to the lack of significant *csgD* expression observed in the majority of isolates tested at temperatures ≥37°C (Römling et al., 1998; Römling et al., 2003; White et al., 2008) there is now ample evidence to show *Salmonella* produces EPSs *in vivo* and that chronic infections are mediated by biofilms. Regarding the effect of temperature on *csgD* expression, further investigations revealed the true complexity of the regulatory network governing *csgD* expression; some postulated that other environmental signals encountered *in vivo* might be able to override the effects of temperature on *csgD* expression (Simm et al., 2014). While the intricacies of such a regulatory hierarchy are still under investigation, numerous research groups have also published evidence of *Salmonella* biofilm formation in various *in vivo* systems. Perhaps most well-documented is the contribution of biofilms to chronic gallbladder carriage of the human-specific *S*. Typhi (Parry et al., 2002; Bhan et al., 2005; Gonzalez-Escobedo et al., 2011; Gunn et al., 2014). As mentioned previously, while infection with *S*. Typhi most commonly results in acute disease (typhoid fever), chronic infections may also develop (Parry et al., 2002; Bhan et al., 2005; Gunn et al., 2014). The majority of these chronic infections are localized to the gallbladder and progression to chronic disease is associated with the presence of gallstones or other gallbladder abnormalities as this host environment provides an appropriate surface for *S*. Typhi to attach and establish robust biofilms (Schioler et al., 1983; Lai et al., 1992). Initial attachment of *S*. Typhi to gallstone cholesterol surfaces is mediated by FliC on flagellar appendages (Crawford et al., 2010a). However, *S*. Typhi can also anchor itself to the gallbladder epithelium, suggesting additional biofilm-forming capabilities *in vivo* (Crawford et al., 2010b; Gonzalez-Escobedo and Gunn, 2013; Marshall et al., 2014). This mechanism of chronic infection has also been recapitulated in mice by intraperitoneal infection of NRAMP^+/+^ mice with *S*. Typhimurium (Crawford et al., 2010b). Importantly, the chronic mouse model of infection involves treatment of the mice for 8 weeks prior to infection with a lithogenic diet to induce gallstone formation, thus providing an optimal environment for *Salmonella* biofilm formation. In this context, biofilms provide *Salmonella* with increased recalcitrance to the harsh gallbladder environment (which includes detergent-like bile salts) and other potential threats such as the host immune system or antibiotic therapy, allowing for long-term survival within the host and prolonged transmissibility *via* intermittent fecal shedding (Jolivet-Gougeon and Bonnaure-Mallet, 2014; González et al., 2018; Hahn and Gunn, 2020; Tsai et al., 2020). Chronic gallstone biofilm carriers can shed *S*. Typhi for at least a year or longer (Gonzalez-Escobedo et al., 2011). This population represents the only known reservoir of *S*. Typhi, with subsequent transmission to others either directly or through contamination of food and water, which is a significant public health challenge for addressing endemic typhoid fever.

The extent to which non-typhoidal *Salmonella* (NTS) serovars utilize biofilm formation during the natural course of infection is less apparent. *In vitro*, numerous NTS isolates exhibit biofilm-like adherence to both human and chicken epithelial cells; however, the effects of biofilm-mediated adherence on virulence were variable (Solano et al., 2001; Ledeboer and Jones, 2005; Ledeboer et al., 2006; Lamprokostopoulou et al., 2010). Whereas *S*. Typhimurium biofilm formation on HT-29 cells was associated with a relative decrease in virulence characterized by reduced invasion and production of pro-inflammatory IL-8, the level of *S*. Enteritidis biofilm formation on HEp-2 and Caco-2 cells was positively correlated with virulence as measured by the extent of epithelial barrier disruption (Solano et al., 2001; Lamprokostopoulou et al., 2010). While the transition to the biofilm phenotype is likely to have pleiotropic effects on the *Salmonella* virulence program, evidence from *in vivo* studies suggests that, overall, biofilm formation is utilized to simultaneously reduce virulence and increase persistence. Within the cecum of newborn chickens, biofilm formation is a key asset for both *S*. Typhimurium and *S*. Pullorum, allowing the bacteria to successfully overcome colonization resistance and establish persistent asymptomatic infection (Sheffield et al., 2009; El Hag et al., 2017). *Salmonella* Typhimurium biofilms have also been implicated in the formation of extracellular aggregates *in vivo*. In the *C. elegans* gut, CsgD-dependent formation of aggregates was associated with relative increases in both bacterial persistence and host survival, a phenotypic shift that likely contributes to the high transmissibility of similar aggregative populations within the cecum and colon of mice (Lam and Monack, 2014; Desai and Kenney, 2019; Desai et al., 2019). While persistent and/or recurrent NTS infections of the human intestine have been documented, reports of cases are limited and do not provide any concrete evidence that would implicate the involvement of biofilms (Musher and Rubenstein, 1973; Kazemi et al., 1974; Buchwald and Blaser, 1984; Marzel et al., 2016; Gal-Mor, 2019). However, antibodies to curli have been detected in the serum of patients recovering from NTS infection and recent work in mice orally infected with *S*. Typhimurium demonstrated the production of curli amyloids in the gastrointestinal tract, suggesting that expression of biofilm genes does occur on some level during human infection (Tursi and Tukel, 2018; Miller et al., 2020).

**Figure 1:**
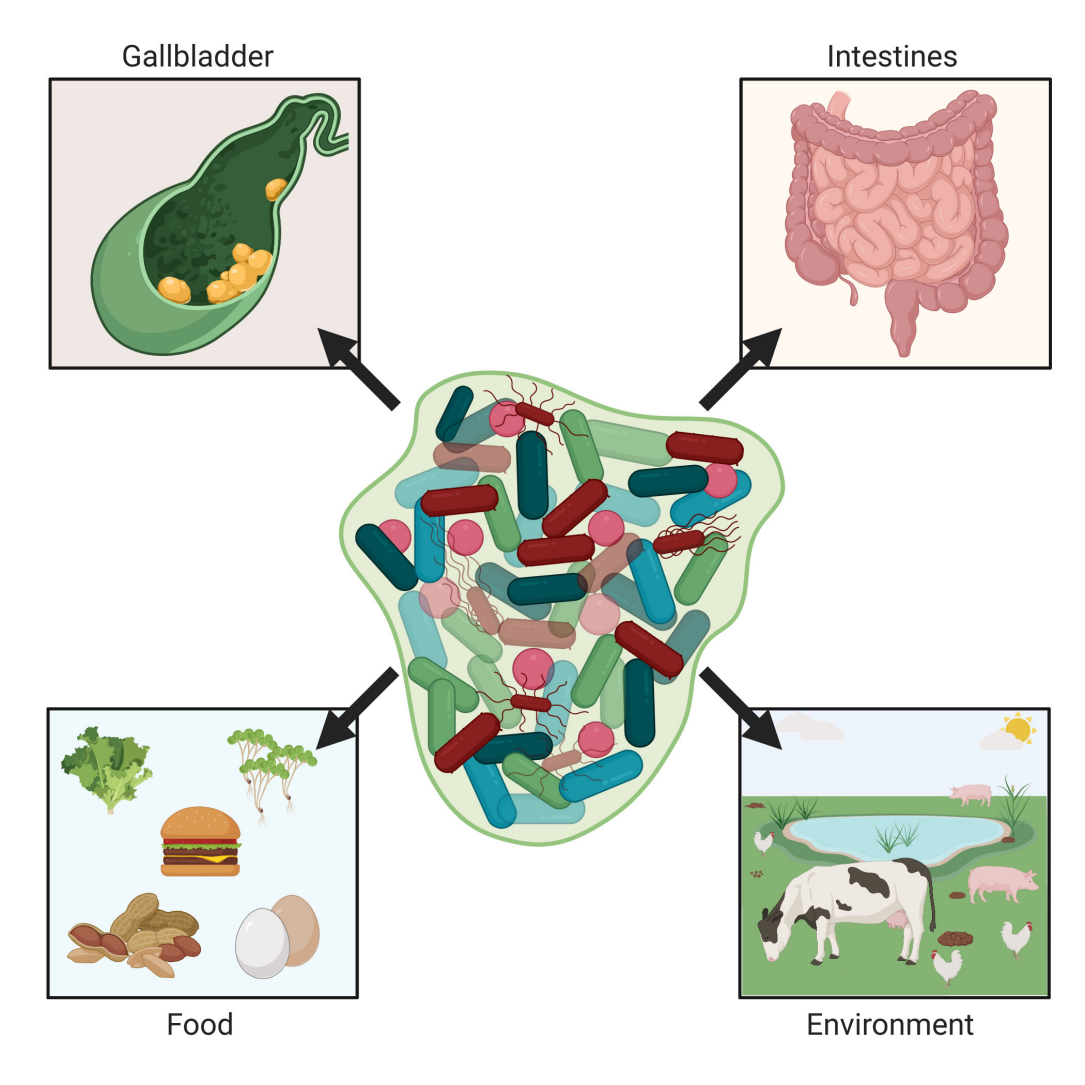
*Salmonella* biofilms in the environment and humans. Non-typhoidal and typhoidal salmonellae are naturally acquired by humans through environmental or food sources. *Salmonella* Typhimurium colonizes and can form biofilms on various types of produce, while *S*. Enteritidis is often found within and on eggs. When within the intestines of various non-human animal species, salmonellae can cause either diarrheal disease or be a non-pathogenic inhabitant (e.g., in the chicken). This can lead to contaminated meat upon animal processing or shedding resulting in contaminated animal feces. Once ingested by the host, *Salmonella* spp. are capable of forming biofilms in the intestines and for *S*. Typhi, after systemically gaining access to the liver, can pass into the gallbladder and form biofilms on cholesterol gallstones. These biofilms within humans facilitate chronic *Salmonella* infection as well as continual shedding of *Salmonella* from the host. Images created with BioRender.com.

In summary, the ability to form a biofilm is a conserved trait found in numerous serovars of *S. enterica*. Biofilm formation allows *Salmonella* to persistently colonize sites both inside and outside of the animal host, ultimately enhancing both bacterial survival and transmission (**Figure 1**).

## *Salmonella* Typhi Early Infection and Invasion of the Gastrointestinal Tract

After ingestion, the first site of infection is typically the small intestine where *S*. Typhi uses its SPI-1 type III secretion system (T3SS-1) to enable penetration of the epithelial layer of the small intestine, typically *via* microfold (M) cells of the Peyer’s patches (Kohbata et al., 1986). *Salmonella* Typhi can be found within the Peyer’s patches 3 to 6 h post infection (Carter and Collins, 1974). This invasion of M cells leads to their destruction, further disrupting the intestinal barrier and promoting increased entry of *Salmonella* (Jones et al., 1994). *Salmonella* is also capable of disrupting the tight junctions found between epithelial cells which further increases permeability (Boyle et al., 2006). Following entry at Peyer’s patches, the primary innate immune response is initiated in resident dendritic cells (DCs) and macrophages. These cells recognize *Salmonella via* the pattern recognition receptors called toll-like receptors (TLRs) and NOD-like receptors (NLRs) that detect pathogen associated molecular patterns (PAMPs), such as lipopolysaccharide (LPS). These PAMPs trigger an immune response through a multitude of TLRs expressed on the surface of immune cells, notably TLR4, TLR5, and TLR9 (Zeng et al., 2003; Lahiri et al., 2010; Rathinam et al., 2019). LPS, particularly the lipid A portion, is recognized by TLR4 on DCs or macrophages. *Salmonella* can adapt *in vivo* to modify its LPS, which promotes immune evasion *via* decreased colonization and pro-inflammatory cytokine production as well as by eliciting a change in the type I interferon response (Kong et al., 2012; Avraham et al., 2015).

*Salmonella* Typhi expresses a polysaccharide capsule with incredible antigenic capability (Parry et al., 2002). The Vi antigen is an important virulence factor of typhoid-inducing *Salmonella* and is encoded on the *Salmonella* pathogenicity island (SPI) SPI-7 (Seth-Smith, 2008). This capsular antigen resists complement deposition and reduces phagocytic death by decreasing PAMP expression (Sharma and Qadri, 2004; Atif et al., 2014; Wangdi et al., 2014). Additionally, Vi antigen and other biofilm exopolysaccharides are advantageous in that they can mask LPS, allowing the pathogen to evade TLR4 recognition by both DCs and macrophages (Wilson et al., 2008). An additional virulence factor important for *S*. Typhi pathogenesis is typhoid toxin, which has been shown to cause DNA damage that can suppress the intestinal inflammatory response, which could potentially increase the frequency of chronic asymptomatic carriers. An additional sequela of chronic colonization of the gallbladder is the development of gallbladder cancer, which could also potentially be related to the typhoid toxin (Del Bel Belluz et al., 2016).

Once recognized by innate immune cells, *Salmonella* is readily phagocytosed by both DCs and macrophages, in which they can reside within a specialized intracellular structure called a *Salmonella* containing vacuole (SCV). DCs infected with *Salmonella* traffic to the gut-draining mesenteric lymph nodes (mLNs) where they present antigen to naïve T cells. Activation of T cells in either the mLNs or Peyer’s patches requires CD11c^+^CCR6^+^ DCs (Salazar-Gonzalez et al., 2006). The mLNs are an important site of infection control, as it is known that mice lacking mLNs demonstrate increased bacterial burdens in systemic tissues following relapse of the primary infection due to increased colonization of the liver and spleen (Voedisch et al., 2009). Once phagocytosed, NLRs recognize bacterial components in the cytosol and trigger an inflammasome response (Ferrand and Ferrero, 2013). The response of the innate immune system leads to downstream effects in the adaptive immune response, which is essential for long term infection control of persistent *Salmonella* infection.

## Adaptive Immune Responses to Intestinal *Salmonella* Typhi Infection

The cell-mediated adaptive immune response, as opposed to the humoral response, is important for cytokine production by T cells that have a significant role in infection control. Both CD4^+^ and CD8^+^ T cells are important during *Salmonella* infection of humans due to their production of IFN-γ (Bao et al., 2000; McSorley et al., 2000). Results from controlled human infection studies have shown that regulatory CD4^+^ T cells (T_regs_) are important in suppressing effector T cells (Clay et al., 2020). The activation of T_regs_ is likely important for balancing the pro-inflammatory response with the suppressive immune response, both of which are potentially damaging to the host if not carefully regulated (Johanns et al., 2010; McArthur et al., 2015). *Salmonella* Typhi genes, such as *tviA*, which encodes a protein involved in Vi antigen synthesis, can interfere with expansion of flagellin-specific CD4^+^ T cells at mucosal sites by reducing the amount of the dominant *Salmonella* antigen FliC; this change in gene expression helps *S*. Typhi to be able to disseminate more rapidly compared to *S*. Typhimurium (Atif et al., 2014). Several other studies highlight that *Salmonella* is capable of ubiquitinating surface major histocompatibility complex II (MHC-II), thereby inducing its degradation; the resulting loss of surface MHCII expression allows the bacterial invaders to evade the CD4^+^ T cell response (Cheminay et al., 2004; Mitchell et al., 2004; Halici et al., 2008; Lapaque et al., 2009; Jackson et al., 2013). A study in 2016 found that CD8^+^ T cells were critical for infection control of *S*. Typhi in humans; the T effector memory and T effector memory CD45RA^+^ cells were the subsets most associated with protection from typhoid-like disease, as those who were protected from typhoidal disease showed higher *S*. Typhi-specific responses at baseline (Fresnay et al., 2016).

The responses seen in both CD4^+^ and CD8^+^ T cells depend largely on initial stimulation. CD4^+^ T cell responses are initiated against soluble antigens, like flagellin, whereas CD8^+^ T cell responses are derived from *S*. Typhi-infected antigen presenting cells, predominantly DCs (McSorley et al., 2002; Jones-Carson et al., 2007; Bobat et al., 2011). In the presence of *S*. Typhi, DCs mature through suicide-cross presentation and are able to induce expansion of *Salmonella*-specific CD8^+^ T cells (Salerno-Goncalves and Sztein, 2009). The relationship between B cells and control of *S*. Typhi infection in humans is unclear. However, it is known that B cells are required to generate antibodies and to prime T cells in the context of *Salmonella* infections. Antibodies, specifically IgM, play a role in typhoidal disease protection and require help from IFN-γ producing CD4^+^ T cells to maintain a memory response (Perez-Shibayama et al., 2014). Furthermore, anti-*Salmonella* serum IgG and secreted IgA (sIgA) are found in patients infected with *S*. Typhi and B cells appear to be important during secondary infections as they may serve as antigen presenting cells to prime the CD4^+^ T cell response (Lopez-Medina et al., 2014).

## Adaptive Immune Responses to Intestinal *Salmonella* Typhimurium Infection

While *S*. Typhi and *S*. Typhimurium share 90% of their genetic sequence, this difference is enough to alter host immune responses (Tang et al., 2013). *Salmonella* Typhimurium is one of the most common serovars of *Salmonella* to cause gastroenteritis infections in humans and is also able to establish typhoid-like infection in mice, providing an insightful infection model for the study of systemic and/or chronic *Salmonella* pathogenesis and immune evasion mechanisms (Santos et al., 2001). *Salmonella* Typhimurium notably lacks the Vi capsule and typhoid toxin expressed by *S*. Typhi, but does have alternative effector proteins enhance immune evasion. Like *S*. Typhi, *S*. Typhimurium is an oral-fecal pathogen and encounters the small intestine first, where it crosses the Peyer’s patches *via* M cells. Resident macrophages and DCs play a role in systemic spread of *S*. Typhimurium through their ability to phagocytose bacteria. Survival and replication of *S*. Typhimurium in SCVs relies on the SPI-2 encoded type III secretion system-2 (T3SS-2), which delivers more than 20 effectors through the SCV membrane into the cytosol of the host cell. Once in the cytosol, effectors can regulate the host environment so as to help the bacteria escape lysosomal degradation (Drecktrah et al., 2006; Figueira and Holden, 2012; Johnson et al., 2018). In mice, these evasion and adaptation mechanisms allow *S*. Typhimurium to survive and disseminate systemically and into the hepatobiliary system. Like *S*. Typhi, *S*. Typhimurium causes apoptotic disruption of the epithelial layer of the small intestine in humans, which is activated through the caspase-8 mediated inflammasome (Hefele et al., 2018). The production of IFN-γ by Natural Killer (NK) cells, especially during the initial stages of infection, helps control the systemic bacterial burden (Kupz et al., 2013; Ingram et al., 2017). Following invasion of the intestines, *S*. Typhimurium prevents DC antigen presentation by avoiding lysosomal degradation and causing DC dysfunction (Tobar et al., 2006; Bueno et al., 2008). Additionally, DCs require TLR stimulation and expression of the chemokine receptor CXCR1 to induce bacterial sampling (Rescigno et al., 2001; Niess et al., 2005). To control and clear *S*. Typhimurium infection in mice, Th1 and Th17 CD4^+^ T cells are important while CD8^+^ T cells may not be necessary in the early stages of the primary response (Lee et al., 2012a). Notably, early depletion of CD8^+^ T cells does not significantly affect splenic bacterial burden (Johanns et al., 2010). Several *Salmonella* proteins including flagellin and SseJ are responsible for inducing the Th1 and Th17 CD4^+^ T cell responses observed during infection, and B cell activation has been found to be required to activate Th1 and Th17 responses (Barr et al., 2010; Lee et al., 2012b). Following *S*. Typhimurium infection in mice, IgM, IgG, and IgA antibodies are produced, and passive transfer of these antibodies confers protection against infection, demonstrating that B cells help protect against secondary infection (Nanton et al., 2012). sIgA predominates in the gastrointestinal tract and aids in obstructing bacterial penetration of the intestinal epithelium while IgG opsonizes bacteria for increased uptake by macrophages (Uppington et al., 2006; Nanton et al., 2012). sIgA is primarily secreted from B cells found in the intestinal lamina propria and prevents bacteria from adhering to the mucosal surface (Mantis and Forbes, 2010).

Typically, *S*. Typhimurium and related NTS strains only cause gastroenteritis in humans. However, there is increasing prevalence of invasive non-typhoidal *Salmonella* (iNTS), particularly in sub-Saharan Africa. One distinct *Salmonella* strain in this region (ST313) appears genetically adapted to cause increased systemic disease in humans. This adaptation results in hyper-dissemination of *Salmonella via* CD11b^+^CCR7^+^ migratory DCs (migDCs) through the lymphatics (Carden et al., 2017). ST313 has evolved other mechanisms to promote invasive disease such as overexpression of *ribB* to avoid detection by Mucosal-associated Invariant T cells by reducing their activation (Preciado-Llanes et al., 2020). SseI, a known immunogen that induces a potent and sustained CD4^+^ T cell response, inhibits the migration of infected DCs (Kurtz et al., 2014; Carden et al., 2017). Notably, migDC-mediated dissemination is increasingly being observed with other *Salmonella* strains such as SL1344, demonstrating that iNTS is becoming more widespread (Carden et al., 2017).

Following the initial infection and transcytosis of M cells, *S*. Typhimurium uses its T3SSs to enter the lamina propria of the small intestine as soon as 8 h post infection (Muller et al., 2012). Approximately 20–40% of bacteria in the lamina propria are found within CD11c^+^CX_3_CR1^high^ macrophages (Muller et al., 2012). Other studies have shown that CD64^+^ macrophages or F4/80^+^CD11b^+^ macrophages are important in the small intestine lamina propria and contribute to T cell activation in intestinal tissue (Carden et al., 2017; Yu et al., 2018). The lamina propria harbors a multitude of immune cell types including B cells, T cells, DCs, NK cells, and macrophages. Breach of this tissue by *Salmonella* triggers a local immune response that contains the infection in this site and prevents further dissemination. However, further breach of this tissue or subversion of immune function can lead to systemic dissemination of iNTS to organs rich in macrophages such as the liver and spleen. Because the liver is a target organ, systemic colonization generally involves the hepatobiliary system, where bacteria can further avoid the immune response and enter a persistent state.

## *Salmonella* Typhi Microbial Pathogenesis and Immune Evasion Within the Hepatobiliary System

Kupffer cells, the liver-resident macrophages, phagocytose *S*. Typhi, and establish SCVs, which help evade immune surveillance (Dougan et al., 2011). From the liver, *S*. Typhi can go on to colonize the biliary tract *via* the ducts or vasculature that connect the liver to the gallbladder. As mentioned previously, studies have shown a strong correlation between the presence of gallstones and the chance of progressing to a chronic carrier state (Schioler et al., 1983).

Although recent studies have provided additional insight into asymptomatic carriage, the mechanisms behind this process in humans are still poorly understood. Information gained from *S*. Typhimurium infection in murine models is vital; however, relevance to human typhoidal infection cannot be automatically assumed. For example, one recent study found that exposure to bile causes an upregulation in *S*. Typhi genes associated with motility, lipid A modulation, and the virulence genes *srfA* and *srfB*. These same genes were significantly downregulated in response to bile in *S*. Typhimurium, highlighting differential responses to physiological changes and the potential mechanism for why these two serovars establish different types of infection (Johnson et al., 2018).

An intriguing finding that requires more investigation is that older females are at higher risk for *S*. Typhi carriage and the subsequent development of gallbladder cancer (Rakic et al., 2014). In a transcriptomic analysis of a chronic typhoid murine model using all female mice, some of the most downregulated genes at 7 and 21 days post infection were hormonal metabolism genes, a finding at odds with earlier studies showing an increase in steroid metabolism genes in the liver after *Salmonella* infection (Antunes et al., 2011; González et al., 2019a). Mice that were fed a lithogenic diet to induce gallstone development also had higher levels of inflammation and *Salmonella-*specific CD4^+^ T cells but were less capable of controlling bacterial infection, possibly because of an overreactive T_reg_ response, which is known to be elevated in females in many inflammatory diseases (González et al., 2019a). A previous group has shown that estradiol can promote production of IFN-γ—an important Th1 cytokine for *Salmonella* clearance—by the immunoregulatory T cell subtype known as invariant natural killer cells (Gourdy et al., 2005). Women are also more susceptible to developing gallstones, and previous studies have shown that treatment with estradiols or progesterone can alter mouse susceptibility to intraperitoneal *S*. Typhimurium challenge (Kita et al., 1989; Stinton and Shaffer, 2012). The human sexual dimorphism seen in chronic *S*. Typhi carriage needs to be explored in depth to better develop informed therapeutics that would decrease the prevalence of asymptomatic spreaders and, ideally, reduce downstream sequalae such as potentially fatal gallbladder malignancies.

## *Salmonella* Typhimurium Microbial Pathogenesis and Immune Evasion of the Hepatobiliary System

As with *S*. Typhi in humans, once disseminated to the liver iNTS in humans or *S*. Typhimurium in mice can take up residence in Kupffer cells, which play a role in maintaining immune homeostasis by triggering T_regs_ and attenuating activation of anti-bacterial inflammatory Th1 CD4^+^ T cells (Rathman et al., 1996; Klugewitz et al., 2002; Breous et al., 2009). Although Kupffer cells are known to prevent bacteria from entering the hepatobiliary system during non-disease states, the immunosuppressive environment of the liver may provide a permissive niche for *S*. Typhimurium. A recent study showed that during persistent infection in resistant mice, *S*. Typhimurium was able to adapt through distinct mutations that were liver- and spleen-specific (Sondberg and Jelsbak, 2016). Further supporting the idea of adaptation, Johnson et al. found that *S*. Typhimurium significantly downregulated many motility genes when grown in the presence of bile, showing that *S*. Typhimurium may establish chronic infection in the liver following localization and adaptation to the otherwise inhospitable environment of continuously flushing bacteriostatic bile salts (Johnson et al., 2018).

From the liver, *S*. Typhimurium can also travel to the gallbladder. During acute infection, *S*. Typhimurium can be found in the gallbladder lumen or tissue within 48 h. Active gallbladder epithelium invasion is followed by proliferative replication in SCVs. An inflammatory response initiated by neutrophils causes tissue damage and sloughing of host epithelial cells, which helps released *S*. Typhimurium invade additional cells (Menendez et al., 2009). As with *S*. Typhi, colonization of the gallbladder by *S*. Typhimurium can result in asymptomatic chronic carriage leading to the unwitting spread of bacteria by seemingly convalescent patients through the fecal-oral route (Sirinavin et al., 2004). Chronic carriage of iNTS is particularly a problem in sub-Saharan Africa (Kingsley et al., 2009).

As discussed above, a robust CD4^+^ Th1 response is essential during the systemic infection phase with *S*. Typhimurium. However, it has also been shown that other adaptive immunity processes are important. Th17 cells produce IL-17 which is needed to recruit neutrophils that aid in controlling infection (Raffatellu et al., 2008). We recently showed that in a biofilm-mediated chronic murine typhoid model, the immune response shifts to a less effective Th2 response by 21 days post infection that was characterized by increased yet ineffective antibody production and increased expression of GATA3, a Th2 transcriptional regulator (González et al., 2019a). Furthermore, the *Salmonella*-specific T cell compartment differs greatly between the lymphoid tissues, such as the mLNs, and the liver (Kurtz et al., 2020). In this case, liver T cells appear to be far more immunosuppressive and permissive of disease compared to lymphoid T cells. They also produced far more of the immunoregulatory cytokine IL-10 compared to lymphoid CD4^+^ T cells, which were more likely to produce IFN-γ. In addition, lack of thymic output prior to persistent *S*. Typhimurium infection results in a loss of control over infection, despite the fact that *Salmonella* specific CD4^+^ T cells are elevated (Goggins et al., 2020). It will be important to explore whether the thymus contributes more T cells to the lymphoid compartment or the hepatobiliary tissue. The compounded virulence and immune evasion techniques of *S*. Typhimurium and *S*. Typhi, along with inappropriate or manipulated immune responses of the host, make them formidable pathogens in both humans and mice.

## Discussion/Summary

**Figure 2:**
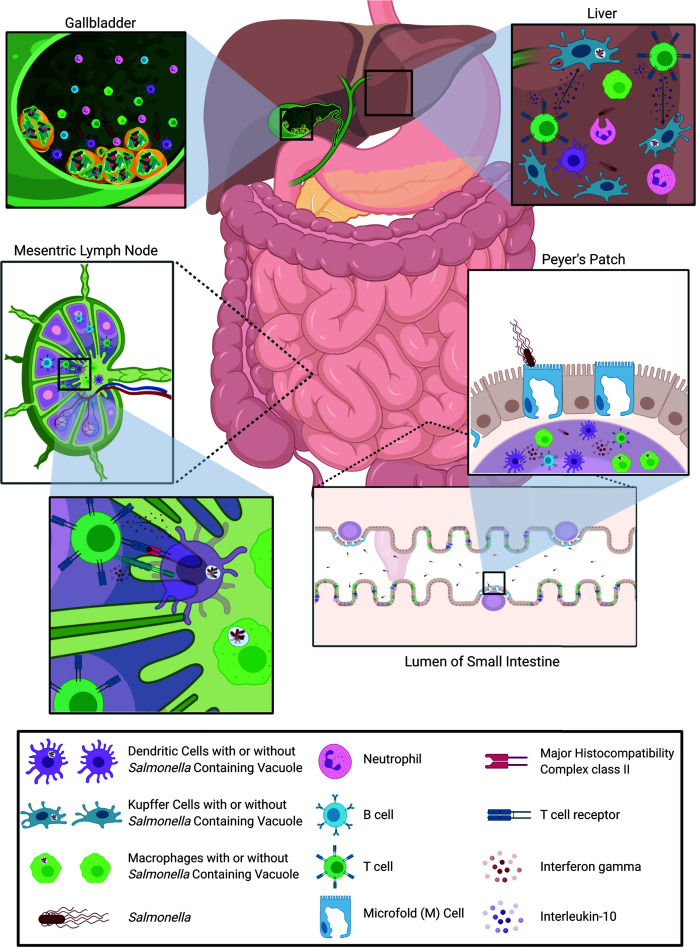
Overview of host immune response to *Salmonella* infection. *Salmonella*, infecting *via* the oral-fecal route, first passes through the stomach and into the lumen of the small intestine. There, *Salmonella* uses its SPI-1 type III secretion system to preferentially transcytose across M cells to gain access to the Peyer’s Patches. Immune cells in the Peyer’s Patches, such as macrophages and dendritic cells, sense *Salmonella via* toll-like receptors and begin to trigger an immune response. Macrophages and dendritic cells then migrate to the gut-draining mesenteric lymph nodes and the spleen (not shown) harboring *Salmonella* antigens as well as whole bacteria in *Salmonella*-containing vacuoles. In the lymph nodes and spleen, dendritic cells and macrophages present antigen on MHC class II to activate *Salmonella*-specific helper T cells which can then subsequently activate *Salmonella*-specific B cells. These adaptive immune cells then traffic back into the intestine to fight infection by releasing the antimicrobial cytokine interferon gamma or neutralizing antibodies. Any *Salmonella* that escape the immune response of the Peyer’s patches and mesenteric lymph nodes or spleen, can travel to, and invade, the liver and subsequently the gallbladder *via* the bile duct. In the liver, Kupffer cells phagocytose *Salmonella* and activate *Salmonella*-specific T cells to produce the anti-inflammatory cytokine interleukin-10, likely preventing bacterial clearance. The immune environment in the gallbladder is less clear, primarily because of the presence of *Salmonella* biofilms on gallstones, which can hinder/alter the immune response. The liver and gallbladder are both sites where the host can experience chronic infection with *Salmonella*. Images created with BioRender.com.

The immune response to *Salmonella* infection is complex and multifaceted and relies on coordination between the early innate response and the subsequent adaptive response. While adaptive immunity to *Salmonella* has been extensively studied in the lymphoid organs (e.g., mLNs and spleen) the response in the intestine and hepatobiliary system, particularly the gallbladder, is less clear (**Figure 2**). It is notable that there are differences in infection and immune responses between the two major *Salmonella* serovars that cause human disease. As summarized in this review, these differences are attributable to differential subversion of the immune response by the bacteria itself, influenced by its planktonic or biofilm lifestyle, as well as where and how the immune system first encounters the bacteria. While progress has been made in our understanding of how *Salmonella* interacts with the host gut and hepatobiliary system, important questions remain regarding these types of interactions:

1. *Does biofilm formation affect the development, function, and subversion of the immune response in the gut and gallbladder?* Our group has already shown that gallstones and chronic Salmonella carriage in the gallbladder recruits both T and B cells to the gallbladder itself (González et al., 2019a). Over time, these immune cells develop a Th2 phenotype in the gallbladder. It remains to be explored whether this Th2 shift is permissive of infection in the gallbladder and thus contributes to persistence. Future studies could examine this question by depleting T or B cells and assessing the respective contributions to gallbladder immunity over time. Surprisingly little is known regarding intestinal immunity during the chronic phases of infection and so these studies could also be extended to the intestine.
2. *How does the presence of gallstones and biofilms regulate persistence and bacterial shedding early and late in infection?* It will also be important to determine whether gallbladder immunity creates an environment that promotes increased bacterial shedding in the feces. It is possible that the immunological shift toward a Th2 phenotype not only permits bacterial persistence, but somehow elicits a greater shedding phenotype, potentially via production of anti-inflammatory cytokines such as IL-10 or IL-4. We have already shown that Salmonella specific T cells in the liver produce IL-10 and are permissive of infection and so extending this to the gallbladder and gut would be an important extension of this work (Kurtz et al., 2020).
3. *What immunological mechanisms allow the gut and hepatobiliary system to become permissive to chronic infection?* While the Th2 shift is fairly generalized, it is not known how Salmonella specific T cells specifically shift over time. As previously discussed, some of these cells may adopt a Treg phenotype, either in the liver, the gallbladder, or the intestinal tissue itself. While this remains to be explored, it would offer a logical explanation for why these tissues appear to be more tolerant of infection compared to other tissues such as the spleen.
4. *Does the immune response in the hepatobiliary system and gut differ between chronic typhoidal infection compared to iNTS or acute infection?* This review touches upon some of the major differences between typhoidal and non-typhoidal Salmonella infection; however, it is not well known whether, or how, the immune responses differ between these two types of infections, especially in the hepatobiliary tract and the intestine. Does chronic typhoidal infection drive a more regulatory immune response and acute infection elicits a more robust antibacterial response? These are important questions that must be addressed in order to answer the final outstanding question below.
5. *How can we eliminate chronic carriage?* Understanding the immune response in the major tissues that harbor bacteria, especially the gut or hepatobiliary system, would greatly aid in the fight to eliminate chronic carriage. The ultimate goal of this understanding would be combatting bacterial carriage, thus eliminating human to human spread of infection. It is possible that using biofilm dispersal agents would allow the immune system adequate access to bacteria ensuring their elimination. Alternatively, a vaccination strategy that preferentially targets the gut, as we have previously shown to be possible using the appropriate adjuvant, might be sufficient to eliminate bacteria (Frederick et al., 2018).

Further studies will better elucidate immunological connections between the gut and hepatobiliary system, particularly during chronic infection, and how this might contribute to *Salmonella* pathogenesis, and how to eliminate it from the human population.

## Author Contributions

JH, MH, SD’S, EV, and JS all wrote sections of the manuscript and edited the final version. JH created the figures. MH referenced the manuscript. JG and JM provided funding for the work and edited the text and figures. All authors contributed to the article and approved the submitted version.

## Funding

The work presented here was funded by grants AI116917, AI099525, AI153752 and AI156328 from the National Institutes of Health to JG and National Institutes of Health grant U01 AI124289 and a grant from the WM Keck Foundation to JM.

## Conflict of Interest

The authors declare that the research was conducted in the absence of any commercial or financial relationships that could be construed as a potential conflict of interest.
